# Sensor‐Based Assessment of Asymmetry in Upper Limb Bradykinesia Among Patients With Parkinson’s Disease and Scans Without Evidence of Dopaminergic Deficit (SWEDD)

**DOI:** 10.1155/padi/6400390

**Published:** 2026-04-01

**Authors:** Do Young Kwon, Junghyuk Ko, Yuri Kwon, Ji-Won Kim

**Affiliations:** ^1^ Department of Neurology, Korea University College of Medicine, Ansan-City, South Korea, korea.ac.kr; ^2^ Division of Mechanical Engineering, College of Engineering, Korea Maritime and Ocean University, Busan, 49112, Republic of Korea, kmou.ac.kr; ^3^ Department of Biomedical Engineering, Konkuk University, Chungju-Si, 27478, Chungcheongbuk-Do, Republic of Korea, konkuk.ac.kr; ^4^ Research Institute of Biomedical Engineering, Konkuk University, Chungju-Si, 27478, Chungcheongbuk-Do, Republic of Korea, konkuk.ac.kr

**Keywords:** asymmetry, finger tapping, forearm rotation, gyro sensor, Parkinson’s disease, SWEDD

## Abstract

**Background:**

Bilateral motor asymmetry is a hallmark feature of Parkinson’s disease (PD). However, few studies have quantitatively examined this feature in patients with scans without evidence of dopaminergic deficit (SWEDD). This study aimed to investigate upper limb asymmetry in bradykinesia among PD and SWEDD patients using gyro sensors, focusing on finger tapping and forearm rotation task.

**Methods:**

We recruited 23 early‐stage, drug‐naïve PD patients, 23 SWEDD patients, and 20 age‐matched healthy controls. We recorded gyro sensor signals during 15‐s finger tapping and forearm rotation tasks. Bilateral asymmetry indices were calculated and compared among groups. In addition, repeated measure analysis of variance (ANOVA) was used to examine the interaction of task and group, and Spearman correlation analysis was conducted with clinical motor scores.

**Results:**

No significant group differences were found during finger tapping. However, during forearm rotation, PD patients exhibited significantly greater asymmetry than SWEDD patients in several indices, including RMS angular velocity, peak angular displacement, peak power, and total power. Task × group interaction effects were significant only in forearm rotation, and asymmetry indices from forearm rotation showed significant correlations with clinical motor scores.

**Conclusions:**

Forearm rotation is a sensitive task for detecting motor asymmetry in PD and for differentiating PD from SWEDD. These results suggest that quantitative motor asymmetry indices using wearable sensors could aid clinicians in the identification of potential SWEDD.

## 1. Introduction

Parkinson’s disease (PD) is a progressive neurodegenerative disorder characterized by motor symptoms such as bradykinesia, rigidity, resting tremor, and postural instability [1]. Bradykinesia is one of the cardinal motor symptoms of PD and is an important therapeutic and diagnostic target [2]. Asymmetric motor symptoms of the bradykinesia persist in most cases [3]. This asymmetry in movement has been widely studied as a potential biomarker for disease diagnosis and progression tracking [4]. Furthermore, asymmetric onset has even been considered as a strong evidence criterion for the diagnosis of PD [5]. Despite extensive research, distinguishing PD from other parkinsonian syndromes remains a clinical challenge.

Approximately 4%–14.7% of patients clinically diagnosed with PD exhibit normal dopamine transporter (DAT) imaging, a condition commonly referred to as scans without evidence of dopaminergic deficit (SWEDD) [6, 7]. SWEDD encompasses a heterogeneous group of disorders with diverse etiologies, including dystonia, fragile X‐associated tremor/ataxia syndrome (FXTAS), neurochemical parkinsonism, and psychogenic parkinsonism [8, 9]. Due to this heterogeneity, characterizing the clinical presentation of SWEDD remains challenging [10, 11]. Moreover, SWEDD patients often demonstrate distinct prognostic trajectories compared to those with PD, typically exhibiting minimal or no disease progression despite occasional responsiveness to levodopa [12]. Differentiating SWEDD from early‐stage PD remains a significant clinical challenge, as both conditions share overlapping motor symptoms [13]. Furthermore, while dopaminergic imaging can aid in diagnosis, its high cost often limits its routine clinical use [14]. A deeper understanding of the motor characteristics of SWEDD is therefore essential to improve diagnostic accuracy and prevent unnecessary treatments and healthcare expenditures.

Several studies have examined bradykinesia asymmetry in patients with PD using clinical assessments such as the Unified PD Rating Scale (UPDRS) and instrumented approaches, including the Grooved Pegboard (GP). Kishore et al. demonstrated that during identical simultaneous bimanual tasks, bradykinesia scores of the most affected limb significantly improved, whereas motor performance of the least affected limb deteriorated compared to unimanual task performance [15]. Sara et al. found that motor symptom asymmetry in PD influences hand performance, with the more affected hand showing significant impairments in precision tasks such as the GP place task [16]. However, there has been limited research on whether a similar asymmetry exists in SWEDD patients. Given that SWEDD is a heterogeneous condition with varying underlying etiologies, it remains unclear whether these patients exhibit the characteristic motor asymmetry observed in PD. Investigating motor asymmetry in SWEDD could provide valuable insights into its pathophysiology and aid in distinguishing it from PD.

Therefore, this study aims to assess bilateral asymmetry in bradykinesia using gyroscopic sensors to objectively quantify movement characteristics in PD and SWEDD patients. By analyzing finger tapping and forearm rotation movements, we investigate whether asymmetry indices can offer insights into PD pathophysiology and aid in its differentiation from SWEDD.

## 2. Methods

A total of 69 participants were enrolled in this study, comprising 23 individuals with SWEDD, 23 drug‐naïve patients diagnosed with early‐stage PD, as well as 20 healthy control participants. To minimize the potential confounding effects of age, participants in all groups were selected to have similar age distributions (control: 65.2 ± 7.4 years; SWEDD: 65.1 ± 11.0 years; PD: 65.5 ± 11.4 years). Furthermore, gender ratios were matched across patient groups, with each group including 8 males and 15 females in the patient group.

The diagnosis of PD was established based on the clinical diagnostic criteria set forth by the UK PD Society Brain Bank. In this study, early‐stage PD was operationally defined as corresponding to modified Hoehn and Yahr Stages 1 or 2 (SWEDD: 1.9 ± 0.4; PD: 1.9 ± 0.4). Motor symptom severity was assessed using Part III of the UPDRS.

To differentiate PD from SWEDD, all participants underwent DAT imaging using fluorinated N‐3‐fluoropropyl‐2‐α‐carboxymethoxy‐3‐α‐(4‐iodophenyl) nortropane (FP‐CIT) positron emission tomography (PET). PET scans were visually interpreted by a board‐certified nuclear medicine specialist and independently re‐evaluated by a second board‐certified nuclear medicine specialist for confirmation. Final diagnostic classification was determined based on concordant visual assessments between the two specialists. Patients presenting with parkinsonian symptoms but with normal DAT binding on FP‐CIT PET were categorized as having SWEDD. Brain MRI was also performed for all patients to rule out other neurological conditions. Individuals with a history of atypical or secondary parkinsonism, major psychiatric or neurological disorders, or dementia were excluded from the study. This study was approved by the Ethics Committee of Korea Hospital (IRB approval number: AS11171), and written informed consent was obtained from all participants prior to their inclusion in the study.

Bradykinesia was quantitatively assessed using a custom gyroscope‐based system (CG‐L53, NEC/Tokin, Japan), previously validated for finger tapping and forearm rotation tasks [17–20]. Tasks were selected from UPDRS Part III, with subjects performing each for 15 s as fast and as widely as possible. Angular velocity was recorded at 250 Hz, and displacement was obtained via numerical integration.

To evaluate asymmetry in motor performance, quantitative indices such as root mean squared (RMS) angular velocity, RMS angular displacement, irregularity of angular velocity, irregularity of angular displacement, peak power, total power, and peak frequency were extracted from sensor signals recorded during finger tapping and forearm rotation tasks. These indices were calculated separately for each hand. RMS angular velocity and RMS angular displacement were used to quantify average movement speed and amplitude, respectively [17–20]. Coefficient of variations (CVs) of angular velocity and displacement served as indicators of movement irregularity, reflecting hesitations or inconsistencies during repeated motion [19, 20]. Peak power and total power represented the main and overall intensities of the frequency domain, while peak frequency indicated the dominant frequency component of the motion signal [17–19]. Bilateral asymmetry was quantified using a normalized ratio that reflects the relative difference between limbs. Specifically, asymmetry was computed by dividing the smaller quantitative parameter value by the larger value between the left and right wrists, and subtracting this ratio from 1. This index ranges from 0 (*perfect symmetry*) to values approaching 1 (*greater asymmetry*), and allows for a direction‐independent assessment of interlimb performance differences.

All statistical analyses were performed using SPSS Version 16.0 for Windows (SPSS Inc., Chicago, IL, USA). To compare bilateral asymmetry indices among the control, SWEDD, and PD groups for each task, one‐way analysis of variance (ANOVA) was conducted for each quantitative index derived from finger tapping and forearm rotation. When significant group effects were identified, post hoc pairwise comparisons with Bonferroni correction were performed. Since multiple comparisons were conducted across several asymmetry indices, the Bonferroni correction was used to control for Type I error. Accordingly, the significance threshold was adjusted to *p* < 0.01 [21].

To examine task‐dependent differences in asymmetry between patient groups, repeated measures ANOVA was additionally performed, including SWEDD and PD patients only, with task as a within‐subject factor and group (SWEDD vs. PD) as a between‐subject factor. The controls were not included in this statistical analysis, as the analysis focused on task‐dependent differences between PD and SWEDD patients. In addition, Spearman correlation analysis was used to examine the relationship between asymmetry indices and UPDRS III score. Statistical significance for both repeated measure ANOVA and correlation analysis was defined as *p* < 0.05.

## 3. Results

Table 1 presents the group differences in quantitative asymmetry indices among the control, SWEDD, and PD groups. In the finger tapping task, asymmetry indices showed no statistically significant group differences in all variables (*p* > 0.01). In contrast, the forearm rotation task revealed significant group differences in some variables. In Table 2, post hoc analyses showed that asymmetry indices in the SWEDD group were comparable to those of healthy controls, while PD patients showed significantly greater asymmetry. Specifically, PD patients exhibited significantly greater asymmetry in RMS angular velocity (*p* < 0.001), peak angular displacement (*p* = 0.005), peak power (*p* = 0.008), and total power (*p* < 0.001) compared to SWEDD patients. To assess the effect of hand dominance, a two‐way repeated measures ANOVA was performed for asymmetry indices that showed significant group differences. Hand dominance showed no significant effects (*p* > 0.05) across most indices, with a marginal effect observed only for peak angular displacement (*p* = 0.013), indicating a limited influence on asymmetry patterns.

**TABLE 1:** Comparison of bilateral asymmetry indices between control, SWEDD, and PD patients during finger tapping and forearm rotation tasks.

Task	Quantitative parameters	Individual groups			ANOVA
		Control (*n* = 20)	SWEDD (*n* = 23)	PD (*n* = 23)	Group difference
		Mean (SD)	Mean (SD)	Mean (SD)	(*p*value)
Finger tapping	RMS angular velocity	0.15 (0.13)	0.23 (0.15)	0.22 (0.15)	0.115
	RMS angular displacement	0.18 (0.15)	0.23 (0.14)	0.20 (0.17)	0.570
	Peak angular velocity	0.13 (0.13)	0.20 (0.12)	0.19 (0.15)	0.209
	Peak angular displacement	0.15 (0.12)	0.20 (0.14)	0.20 (0.15)	0.426
	Irregularity of angular velocity	0.35 (0.16)	0.27 (0.20)	0.24 (0.18)	0.128
	Irregularity of angular displacement	0.33 (0.20)	0.38 (0.18)	0.31 (0.13)	0.302
	Peak power	0.31 (0.21)	0.49 (0.28)	0.46 (0.24)	0.045
	Total power	0.26 (0.20)	0.39 (0.21)	0.37 (0.21)	0.076
	Peak frequency	0.13 (0.14)	0.16 (0.16)	0.11 (0.09)	0.394

Forearm rotation	RMS angular velocity	0.11 (0.08)	0.14 (0.11)	0.29 (0.18)	**p < 0.001**
	RMS angular displacement	0.17 (0.11)	0.14 (0.12)	0.25 (0.18)	0.031
	Peak angular velocity	0.14 (0.11)	0.19 (0.14)	0.26 (0.20)	0.040
	Peak angular displacement	0.15 (0.10)	0.13 (0.09)	0.25 (0.16)	**0.004**
	Irregularity of angular velocity	0.34 (0.19)	0.31 (0.23)	0.30 (0.20)	0.878
	Irregularity of angular displacement	0.34 (0.23)	0.34 (0.22)	0.29 (0.21)	0.677
	Peak power	0.39 (0.19)	0.31 (0.06)	0.56 (0.28)	**0.009**
	Total power	0.20 (0.13)	0.25 (0.18)	0.46 (0.23)	**p < 0.001**
	Peak frequency	0.11 (0.11)	0.13 (0.13)	0.16 (0.13)	0.421

*Note:* Bold values indicate statistically significant group differences (*p* < 0.05).

**TABLE 2:** Results of post hoc analyses comparing SWEDD with control and PD.

		**Control vs. SWEDD**	**SWEDD vs. PD**
		***p* value**	***p* value**

Forearm rotation	RMS angular velocity	0.764	**p < 0.001**
	Peak angular displacement	0.795	**0.005**
	Peak power	0.623	**0.008**
	Total power	0.724	**p < 0.001**

*Note:* Bold values indicate statistically significant group differences (*p* < 0.05).

**FIGURE 1:**
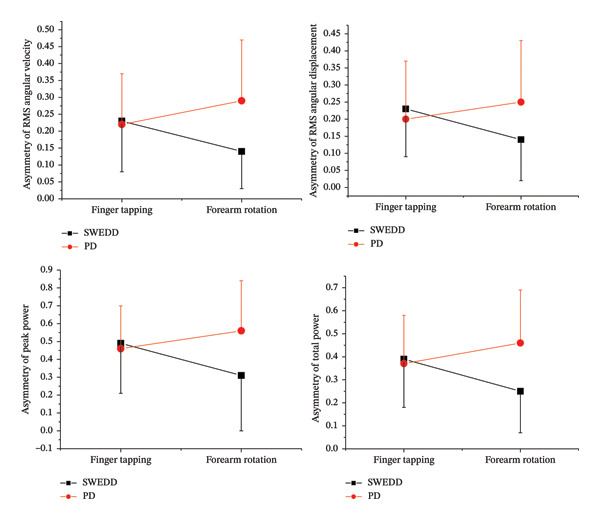
RMS angular velocity and RMS angle in controls, SWEDD patients, and PD patients (^∗^ *p* < 0.05, ^∗∗^ *p* < 0.01, and ^∗∗∗^ *p* < 0.001).

Table 3 summarizes the effects of the task and the interaction between task and group. No significant main effects were found in all bilateral asymmetry indices (all *p* > 0.05). However, a significant interaction effect between task and group was found for RMS angular velocity (*p* = 0.006), RMS angular displacement (*p* = 0.033), peak power (*p* = 0.015), and total power (*p* = 0.005). These interaction effects are further illustrated in Figure 1. The forearm rotation task elicited markedly different responses in SWEDD and PD patients. While PD patients showed increased asymmetry during forearm rotation compared to finger tapping, SWEDD patients tended to exhibit reduced asymmetry in the same task. In contrast, during finger tapping, asymmetry remained relatively similar between the PD and SWEDD groups.

**TABLE 3:** Repeated measures ANOVA results for bilateral asymmetry indices during finger tapping and forearm rotation tasks.

Bilateral asymmetry indices	Task effect	Interaction of task and group
	*p*value	*p*value
RMS angular velocity	0.685	**0.006**
RMS angular displacement	0.529	**0.033**
Peak angular velocity	0.298	0.147
Peak angular displacement	0.792	0.057
Irregularity of angular velocity	0.276	0.870
Irregularity of angular displacement	0.135	0.715
Peak power	0.473	**0.015**
Total power	0.508	**0.005**
Peak frequency	0.690	0.071

*Note:* Bold values indicate statistical significance (*p* < 0.05).

Table 4 reports correlation analyses between bilateral asymmetry indices and UPDRS III scores. Overall, the SWEDD and PD groups showed limited associations between asymmetry measures and UPDRS III scores. However, when combining the two groups, significant correlations were observed between UPDRS III and several asymmetry indices during forearm rotation, including RMS angular velocity (*r* = 0.461, *p* = 0.002), RMS angular displacement (*r* = 0.364, *p* = 0.018), peak angular displacement (*r* = 0.375, *p* = 0.015), total power (*r* = 0.461, *p* = 0.002), peak power (*r* = 0.476, *p* = 0.001), and peak frequency (*r* = 0.442, *p* = 0.003). In contrast, no significant correlations were observed in the SWEDD group across tasks.

**TABLE 4:** Correlation between bilateral asymmetry indices and UPDRS III scores in SWEDD, PD, and combined groups.

Task	Quantitative parameters	Individual groups		
		SWEDD (*n* = 23)	PD (*n* = 23)	SWEDD + PD (*n* = 46)
		Correlation coefficient (significance)	Correlation coefficient (significance)	Correlation coefficient (significance)
Finger tapping	RMS angular velocity	0.115 (0.630)	**0.432 (0.044)**	0.263 (0.092)
	RMS angular displacement	0.007 (0.977)	0.353 (0.107)	0.190 (0.229)
	Peak angular velocity	0.011 (0.965)	0.363 (0.096)	0.206 (0.190)
	Peak angular displacement	0.202 (0.394)	0.336 (0.127)	0.241 (0.123)
	Irregularity of angular velocity	−0.360 (0.119)	−0.110 (0.625)	−0.237 (0.130)
	Irregularity of angular displacement	−0.073 (0.761)	0.134 (0.551)	−0.022 (0.888)
	Peak power	0.161 (0.499)	0.407 (0.060)	0.220 (0.162)
	Total power	0.106 (0.657)	0.414 (0.056)	0.263 (0.092)
	Peak frequency	0.075 (0.753)	−0.215 (0.337)	−0.065 (0.683)

Forearm rotation	RMS angular velocity	0.388 (0.091)	0.256 (0.249)	**0.461 (0.002)**
	RMS angular displacement	0.293 (0.210)	0.190 (0.397)	**0.364 (0.018)**
	Peak angular velocity	−0.125 (0.598)	0.311 (0.159)	0.261 (0.095)
	Peak angular displacement	0.167 (0.482)	0.249 (0.265)	**0.375 (0.015)**
	Irregularity of angular velocity	0.362 (0.117)	−0.082 (0.716)	0.058 (0.714)
	Irregularity of angular displacement	0.405 (0.077)	0.060 (0.791)	0.107 (0.502)
	Peak power	0.328 (0.158)	0.383 (0.079)	**0.476 (0.001)**
	Total power	0.388 (0.091)	0.256 (0.249)	**0.461 (0.002)**
	Peak frequency	0.308 (0.186)	0.567 (0.006)	**0.442 (0.003)**

*Note:* Bold values indicate statistically significant correlations (*p* < 0.05).

## 4. Discussion

This study aimed to compare bilateral motor asymmetry between SWEDD and PD patients using quantitative asymmetry indices derived from finger tapping and forearm rotation tasks. This study also investigated the correlation between asymmetry indices and clinical motor scores. The results revealed task‐specific group differences in several asymmetry indices and highlighted forearm rotation as a more sensitive task for differentiating PD from SWEDD. Specifically, the interaction effects between task and group were observed, and forearm rotation revealed significantly greater asymmetry in the PD group than in the SWEDD group across several asymmetry indices. Moreover, when PD and SWEDD groups were combined, several asymmetry measures during forearm rotation showed significant correlations with motor severity.

Forearm rotation task elicited significant group differences between PD and SWEDD patients in several asymmetry indices, including RMS angular velocity, peak angular displacement, peak, and total power (Tables 1 and 2). These results indicate that PD patients exhibit greater asymmetry in forearm movement speed, angular range, the intensity of the main forearm movement component, and the total signal power of forearm movements compared to SWEDD patients. In contrast, no significant group differences were observed in any asymmetry indices. These results suggest that the forearm rotation task is more sensitive than the finger tapping task for the distinction of SWEDD from PD. Forearm rotation, specifically pronation and supination, is a multijoint movement involving both the proximal and distal radioulnar joints. In addition, it is a coordinated task that requires larger ranges of motion, greater activation of proximal musculature, and greater demands on interlimb coordination compared to finger tapping. These characteristics may accentuate subtle motor deficits, particularly in individuals with asymmetric basal ganglia dysfunction, such as those with PD. In contrast, SWEDD patients, who by definition lack significant presynaptic dopaminergic loss, demonstrated relatively symmetric performance. Notably, the forearm rotation task could be a useful clinical tool for effectively differentiating between neurodegenerative and non‐neurodegenerative parkinsonism. In addition, while asymmetry during finger tapping was similarly increased in both PD and SWEDD patients compared to healthy controls, asymmetry indices in the SWEDD group during the forearm rotation task did not differ significantly from those of healthy controls, suggesting task‐specific preservation of motor symmetry in SWEDD.

The interaction effect between task and group observed in the repeated measures ANOVA further supports the task‐dependent characteristics of motor asymmetry (Table 3). Significant interaction effects were found in several indices, including RMS angular velocity, RMS angular displacement, peak power, and total power. Notably, asymmetry in PD patients became more pronounced during the forearm rotation task compared to finger tapping, whereas SWEDD patients showed the opposite trend, with reduced asymmetry during forearm rotation (Figure 1). These findings suggest that the type of clinical task plays a crucial role in revealing group‐specific motor asymmetry, with forearm rotation being more sensitive in differentiating between parkinsonian pathology and atypical cases such as SWEDD. The lack of group differences during finger tapping may reflect the simplicity of the task, which requires relatively limited joint movement and minimal neural demands. As a result, both SWEDD and PD patients were able to perform finger tapping with relatively similar asymmetry. In contrast, the forearm rotation task imposes greater biomechanical and neural demands, which may unmask pathological asymmetry in PD patients. Interestingly, although SWEDD patients are often reported to exhibit some degree of asymmetry in clinical examinations, reduced asymmetry was observed when the forearm rotation task was quantitatively evaluated using gyro sensors in this study. One possible explanation is that clinical assessment reflects overall motor presentation through observational ratings, which may be influenced by subjective judgment. In contrast, IMU‐based assessment of the forearm rotation task enables continuous and objective quantification of movement, allowing more precise detection of true motor output symmetry. Consistent with this interpretation, standard UPDRS clinical ratings did not reveal significant group differences between PD and SWEDD patients for either finger tapping (*p* = 0.842) or forearm rotation (*p* = 0.317), whereas IMU‐based analysis identified significant group differences during the forearm rotation task. Taken together, these findings indicate that task selection and quantitative measurement are critical for detecting clinically meaningful motor asymmetry and suggest that IMU‐based assessment of forearm rotation may provide complementary diagnostic value beyond standard clinical ratings in differentiating PD from SWEDD.

Moreover, correlation analysis revealed that asymmetry indices during forearm rotation, but not finger tapping, were significantly correlated with UPDRS III scores when SWEDD and PD patients were analyzed together. Specifically, RMS angular velocity, angular displacement, peak angular displacement, and frequency domain indices showed significant correlations, reinforcing their utility in motor assessment. This indicates that forearm rotation is not only effective for distinguishing between SWEDD and PD patients but also useful for assessing the severity of motor symptoms more broadly. The increased biomechanical and coordination demands of forearm rotation may make it more sensitive to detecting motor deficits related to disease severity, whereas finger tapping may be too simple to unmask subtle variations in motor performance.

Recent studies have sought to characterize the clinical profiles of SWEDD patients, primarily focusing on tremor and gait abnormalities. For instance, Schwingenschuh et al. [11] noted a difference in tremor characteristics, with PD patients showing predominantly resting tremor, while SWEDD patients exhibited postural tremor often accompanied by dystonic features. Similarly, some studies reported that SWEDD patients demonstrated relatively normal gait patterns, including trunk and elbow posture, stride length variability, and bilateral step phase coordination, in contrast to the pronounced gait impairments observed in PD patients [13]. Despite these efforts, relatively few studies have investigated bradykinesia, which is a cardinal symptom of PD and an important biomarker in its diagnosis and treatment [14]. Although Schwingenschuh et al. [11] included clinical comparisons of bradykinesia, their analysis relied primarily on clinical rating scales and lacked quantitative assessment. A more recent study attempted to address this by using gyro sensors to compare upper limb bradykinesia between PD and SWEDD patients [20]. While this study offered meaningful insights into bradykinesia, it mainly focused on overall task performance and unilateral movement features, without directly assessing bilateral motor asymmetry. The current study builds upon and advances prior research by introducing quantitative asymmetry indices derived from both finger tapping and forearm rotation tasks. By focusing on bilateral asymmetry, this study provides a novel dimension to understanding motor differences between PD and SWEDD. As motor asymmetry is a clinical feature of early‐stage PD, quantifying asymmetry, particularly in forearm rotation, provides a valuable metric for distinguishing between these two groups.

Despite its strengths, this study has several limitations. The sample size was relatively small, which may limit the generalizability of the findings. Also, the cross‐sectional design precludes any conclusions about the longitudinal progression of motor asymmetry in PD and SWEDD patients. Future studies with longitudinal follow‐up are needed to assess whether these asymmetry patterns change over time.

In conclusion, this study demonstrated that bilateral motor asymmetry, particularly during forearm rotation, provides meaningful insight into differentiating PD from SWEDD patients. While finger tapping did not reveal significant group differences, forearm rotation elicited pronounced asymmetry in PD patients, but not in SWEDD patients. Moreover, asymmetry indices during forearm rotation were significantly associated with the clinical motor severity score. These findings highlight the importance of task‐specific, asymmetry‐focused analysis using wearable sensors as a complementary approach for early and objective differentiation of parkinsonian syndromes.

## Author Contributions

All authors contributed significantly to the study.

Research project: Do Young Kwon, Junghyuk Ko, and Ji‐Won Kim.

Data analysis: Junghyuk Ko and Ji‐Won Kim.

Statistical analysis: Yuri Kwon and Junghyuk Ko.

Manuscript writing: Ji‐Won Kim and Do Young Kwon.

## Funding

This study was supported by the National Research Foundation, 2022R1I1A3065537.

## Disclosure

This paper has not been published or submitted for publication elsewhere. This paper is not under simultaneous consideration by another journal. The funding agency had no role in the design, conduct, or interpretation of this study. All authors are in agreement with the content of the manuscript. Do Young Kwon, Junghyuk Ko, Yuri Kwon, and Ji‐Won Kim have nothing to declare regarding financial disclosures.

## Conflicts of Interest

The authors declare no conflicts of interest.

## Data Availability

Research data are not shared.
